# MicroRNA-16-1-3p Represses Breast Tumor Growth and Metastasis by Inhibiting PGK1-Mediated Warburg Effect

**DOI:** 10.3389/fcell.2020.615154

**Published:** 2020-12-03

**Authors:** Tianxing Ye, Yingchun Liang, Deyu Zhang, Xuewu Zhang

**Affiliations:** ^1^College of Medicine, Yanbian University, Yanji, China; ^2^Department of Medical Molecular Biology, Beijing Institute of Biotechnology, Collaborative Innovation Center for Cancer Medicine, Beijing, China

**Keywords:** the Warburg effect, PGK1, miR-16-1-3p, cell proliferation, metastasis

## Abstract

The Warburg effect (aerobic glycolysis) is a hallmark of cancer and is becoming a promising target for diagnosis and therapy. Phosphoglycerate kinase 1 (PGK1) is the first adenosine triphosphate (ATP)-generating glycolytic enzyme in the aerobic glycolysis pathway and plays an important role in cancer development and progression. However, how microRNAs (miRNAs) regulate PGK1-mediated aerobic glycolysis remains unknown. Here, we show that miR-16-1-3p inhibits PGK1 expression by directly targeting its 3′-untranslated region. Through inhibition of PGK1, miR-16-1-3p suppressed aerobic glycolysis by decreasing glucose uptake, lactate and ATP production, and extracellular acidification rate, and increasing oxygen consumption rate in breast cancer cells. Aerobic glycolysis regulated by the miR-16-1-3p/PGK1 axis is critical for modulating breast cancer cell proliferation, migration, invasion and metastasis *in vitro* and *in vivo*. In breast cancer patients, miR-16-1-3p expression is negatively correlated with PGK1 expression and breast cancer lung metastasis. Our findings provide clues regarding the role of miR-16-1-3p as a tumor suppressor in breast cancer through PGK1 suppression. Targeting PGK1 through miR-16-1-3p could be a promising strategy for breast cancer therapy.

## Introduction

The Warburg effect (also known as aerobic glycolysis) is a phenomenon predominantly observed in tumor cells which give priority to glycolysis to provide energy even when oxygen is sufficient (Schwartz et al., 2017; Tekade and Sun, 2017; Lu, 2019; Pereira-Nunes et al., 2020). Under aerobic condition, more glucose is consumed by tumor cells compared to normal cells, and a large amount of lactate is produced as a metabolite, providing an acidic environment suitable for tumor growth and metastasis. Targeting the Warburg effect is becoming a new strategy for cancer therapy. Phosphoglycerate kinase 1 (PGK1) is one of the key enzymes in the Warburg effect (He et al., 2019; Qian et al., 2019). During glycolysis, PGK1 catalyzes the reversible transfer of the high-energy phosphate group from 1,3-bisphosphoglycerate (1,3-BPG) to ADP, producing 3-phosphoglycerate (3-PG) and adenosine triphosphate (ATP). At the same time, PGK1 promotes glucose uptake and lactate production in cancer cells. Many studies show that PGK1 is highly expressed in various cancers, such as breast cancer, liver cancer, and colon cancer (Ahmad et al., 2013; Hu et al., 2017; Fu et al., 2018). High expression of PGK1 is associated with poor prognosis of cancer patients. PGK1 expression is affected by some transcription factors, such as hypoxia-inducible factor 1α (HIF1α) and sine oculis homeobox 1 (SIX1; Meijer et al., 2012; Li et al., 2018; Nagao et al., 2019). Moreover, phosphorylation and acetylation of PGK1 also regulate tumorigenesis (Hu et al., 2017; Zhang Y. et al., 2018). Thus, PGK1 inhibition with small interfering RNAs (siRNAs) or other approaches may provide a new strategy for cancer treatment.

MicroRNAs (miRNAs) are small, non-coding RNA, typically 18–25 bases in length (Dykxhoorn, 2010; Mulrane et al., 2013; Shen and Hung, 2015; Adams et al., 2017). MicroRNAs usually bind to the 3′-untranslated region (UTR) of mRNA to silence its expression at the post-transcriptional level. Many studies have shown that miRNAs are involved in cancer cell proliferation, invasion and metastasis. Recently, miRNA-450b-3p, miRNA-548c-5p, miRNA-215-5p, and miRNA-6869-5p have been shown to directly target PGK1 3′-UTR and inhibit PGK1 expression (Chen et al., 2019, 2020; Ge et al., 2019; Wang et al., 2020). These miRNAs can regulate cancer cell proliferation, migration and/or invasion. However, whether these miRNAs regulates the Warburg effect via PGK1 remains unclear.

MiRNA-16-1-3p has been reported to act as a tumor suppressor in osteosarcoma (Maximov et al., 2019), non-small cell lung cancer (NSCLC; Feng et al., 2018) and gastric cancer (Wang et al., 2017). However, the function of miRNA-16-1-3p in breast cancer remains unclear. In this study, we show that miRNA-16-1-3p represses aerobic glycolysis in breast cancer cells, leading to inhibition of cancer cell proliferation, migration, invasion, and metastasis *in vitro* and *in vivo*. Mechanistically, miR-16-1-3p inhibits PGK1 expression by directly targeting its 3′-UTR, and represses breast cancer cell growth and metastasis by inhibiting PGK1-mediated Warburg effect.

## Materials and Methods

### Cell Lines, Plasmids, Lentivirus, and Reagents

The human breast cancer cell lines ZR75-1 and MDA-MB-231 and the human embryonic kidney cell line HEK293T were purchased from the American Type Culture Collection. Firefly luciferase-labeled MDA-MB-231 cells were a gift from Professor Yongfeng Shang of Peking University (China). These cell lines were testified without mycoplasma contamination. The PCR amplified fragment was inserted into pcDNA3.0 (Invitrogen) to obtain a PGK1 expression vector. The primers for PGK1 were 5′-CGGGATCCATGTCGCTTTCTAACAAGCTG AC-3′ (forward) and 5′-GCTCTAGACTAAATATTGCTGAGAG CATCC-3′ (reverse) (The underlined part represents the restriction sites). The wild type and mutated miR-16-1-3p putative targets on PGK1 3′-UTR were cloned into pmir-GLO dual luciferase miRNA target expression vector (Promega). Recombinant PCR was performed to generate mutations. The lentiviral vectors that express PGK1 short hairpin RNA (shRNA) were made by cloning PCR-amplified PGK1 shRNA fragment into pSIH-H1-Puro (System Biosciences). The target sequence of PGK1 shRNA was 5′-GUCCAAAGCUGAAGAAUTT-3′. Lentiviruses were produced by cotransfection of HEK293T cells with recombinant lentivirus vectors and pPACK Packaging Plasmid Mix (System Biosciences) using Megatran reagent (Origene), and were used to infect breast cancer cells according to the manufacturers′ instructions. The successfully infected cells were selected with 1 μg/ml puromycin to generate stably cell lines. MiR-16-1-3p mimics and miR-16-1-3p inhibitor were purchased from GenePharma (Jiangsu, China). The sequence of the miR-16-1-3p inhibitor is 5′-UCAG CAGCACAGUUAAUACUGG-3′. The reagents for transfection of plasmids and miRNAs were Lipofectamine 3000 reagent and Lipofectamine RNAiMAX (Invitrogen), respectively. Cells were transfected according to the manufacturers’ protocols. Anti-PGK1 antibody was purchased from Proteintech and anti-α-tubulin antibody was purchased from Santa Cruz Biotechnology.

### Luciferase Reporter Gene Activity

Luciferase reporter assays were carried out according to the manufacturer’s instructions (Promega). Briefly, cells were seeded in 24-well plates. After 24 h, cells were transfected with the wild-type or mutated PGK1 3′-UTR reporter and miR-16-1-3p mimics using Lipofectamine 3000. Twenty four hours after transfection, the cells were harvested and analyzed for luciferase activities according to the manufacture’s instruction (Promega).

### Revere Transcription-Quantitative Polymerase Chain Reaction (RT-qPCR)

Total RNA, including miRNAs, was extracted from cultured cells using the miRcute miRNA Isolation Kit (Tiangen). The miRcute miRNA First-Strand cDNA Synthesis Kit (Tiangen) was used to reverse transcribe the target miRNA into cDNA. For real-time PCR analysis, the primers for PGK1 were 5′-ATGTCGCTTTCTAACAAGCTGA-3′ (forward) and 5′-GCGGAGGTTCTCCAGCA-3′ (reverse). The control primers (α-tubulin) for PGK1 were 5′-CCAAGCTGGAGTTCTCTA-3′ (forward) and 5′-CAATCAGAGTGCTCCAGG-3′ (reverse). The primers for miR-16-1-3p were 5′-GGGGCCAGTATTAACTGT-3′ (forward) and 5′-TGCGTGTCGTGGAGTC-3′ (reverse). The control primers (U6) for miRNAs were 5′-CGCGCTTCGGCA GCACATATACT-3′ (forward) and 5′-ACGCTTCACGAATT TGCGTGTC-3′ (reverse). The relative fold expression of the target, normalized to the corresponding control, was calculated by the comparative Ct methods.

### Cell Proliferation, Migration, and Invasion

Cell proliferation was examined by a CCK-8 Kit according to the manufacturer’s instructions (Dojindo). Cell migration was determined by wound healing assays. Briefly, transfected cells grown to 90% in six-well plates were scratched via a 200 μl pipette tip to create the wound followed by washing detached cells with PBS. The cells were cultured for 16 h to allow wound healing. The wound healing rates were calculated, and compared to the width at 0 h. Cell invasion assay was carried out with Matrigel Invasion Chambers according to the manufacturer’s instructions (BD Biosciences). Briefly, transfected cells were placed on the upper surface of the transwell insert. After 16 h, the invasive cells were fixed with 4% paraformaldehyde and stained with 0.5% crystal violet. The number of invasive cells were counted in five randomly selected microscope visions and photographed.

### Assays of Glucose Uptake and Production of Lactate and ATP

Glucose Uptake Colorimetric Assay Kit, Lactate Assay Kit II and ATP Colorimetric Assay Kit were used to measure glucose uptake and production of lactate and ATP according to the manufacturer’s instructions (Biovision). For glucose uptake colorimetric assay, cells were seeded at a density of 1500 cells per well in a 96-well plate. The cells were starved for glucose by preincubating with 100 μl Krebs-Ringer-Phosphate-HEPES (KRPH) buffer containing 2% BSA for 40 min. Ten microliters of 10 mM 2-DG was added and the cells incubated for 20 min. For lactate and ATP assays, one million cells were homogenized in 100 μl corresponding assay buffer provided by the kits. The homogenized cells were centrifuged, and the soluble fraction was analyzed.

### Assays of Extracellular Acidification Rate and Oxygen Consumption Rate

The extracellular acidification rate (ECAR) and cellular oxygen consumption rate (OCR) were assessed using the Seahorse XFe 96 Extracellular Flux Analyzer (Seahorse Bioscience) according to the manufacturer’s instructions. ECAR and OCR were determined using Seahorse XFe Glycolysis Stress Test Kit and Seahorse XF Cell Mito Stress Test Kit, respectively. Briefly, 1 × 10^4^ cells per well were seeded into a Seahorse XFe 96 cell culture microplate. After baseline measurements, for ECAR, glucose, the oxidative phosphorylation inhibitor oligomycin, and the glycolytic inhibitor 2-DG were sequentially injected into each well at indicated time points; and for OCR, oligomycin, the reversible inhibitor of oxidative phosphorylation FCCP (p-trifluoromethoxy carbonyl cyanide phenylhydrazone), and the mitochondrial complex I inhibitor rotenone plus the mitochondrial complex III inhibitor antimycin A (Rote/AA) were sequentially injected. Data were analyzed by Seahorse XFe 96 Wave software. OCR is indicated in pmols/minute and ECAR in mpH/minute.

### Analysis of Tumor Growth and Metastasis in Nude Mice

We performed animal experiments with the approval of the Institutional Animal Care Committee of the Beijing Institute of Biotechnology. A total of 1 × 10^7^ MDA-MB-231 cells harboring different constructs were subcutaneously inoculated into second mammary fat pad on the right side of nude mice. Tumor size was examined at a specified time using a caliper. The tumor volume is calculated according to the following formula: volume = (longest diameter × shortest diameter^2^)/2. The mice were euthanized at the designated time. Excised tumors were frozen in liquid nitrogen for further study.

For lung metastasis analysis, 1 × 10^7^ MDA-MB-231 cells stably expressing PGK1 shRNA or control shRNA with firefly luciferase labels were treated for three days with antagomiR-16-1-3p (miR-16-1-3p inhibitor) (1 μmol) or antagomiR-NC (scramble) (1 μmol), a negative control. The treated cells were collected and 1 × 10^6^ cells were injected into the lateral tail vein of each BALB/c female mouse. After the indicated times, the mice were imaged using the IVIS200 imaging system (Xenogen Corporation, Alameda, CA, United States). After euthanasia, all lungs were excised for metastatic foci assessment.

### Clinical Samples, MiRNA *in situ* Hybridization and Immunohistochemical Staining

Ninety one human breast cancer samples were obtained from the Chinese PLA General Hospital, with the informed consent of patients and with approval for experiments from the hospital. None of the breast cancer patients had received any chemotherapy prior to surgery. All patients were female with 31-74 years of age (mean age: 51.9 years).

For examination of miR-16-1-3p expression levels in breast cancer samples, miRNA *in situ* hybridization (MISH) was performed. Slides were hybridized with 200 nM of 5′-digoxigenin (DIG) LNA-modified- miR-16-1-3p (GenePharma, China), and then incubated with anti-DIG-horse reddish peroxidase (HRP) (Zhongshan Biotech). The miRNA signal was amplified by TSA Plus Cyanine 3 system (Perkin Elmer). The sequences complementary to miR-16-1-3p were 5′-TCAGCAGCACAGT TAATACTGG-3′. A U6 probe 5′-GAACGCTTCACGAATT TGCGTGTCATCCTTGCGCA-3′ was used as a positive control. A scramble probe 5′-GTGTAACACGTCTATACGCCCA-3′ was used as a negative control. To detect miR-16-1-3p expression, immunohistochemical staining (IHC) of formalin-fixed paraffin-embedded breast cancer samples was performed as described previously (Zhang et al., 2005). Rabbit anti-PGK1 was used at dilutions of 1:200 as primary antibodies for IHC. The expression of miR-16-1-3p and PGK1 was assessed by H score method. H score was generated by multiplying the percentage of stained cells (0–100%) by the intensity of the staining (low, 1+; medium, 2+; strong, 3+).

### Statistics Analysis

All *in vitro* experiments were performed in triplicate and repeated three times unless otherwise indicated. Statistical significance in cell line experiments was determined by two-tailed Student’s *t*-test (two groups) or the ANOVA-Dunnett test (more than two groups). Estimation of disease-free survival was performed using the Kaplan–Meier method, and differences between survival curves were determined with the log-rank test. Statistical analysis was performed using SPSS 17.0 statistical software package. The correlation between the expression of miR-16-1-3p and PGK1 was calculated by Spearman rank correlation analysis using GraphPad Prism 7. *P* values of less than 0.05 were considered statistically significant.

## Results

### Identification of MiR-16-1-3p as a Direct Upstream Regulator of PGK1

**FIGURE 1:**
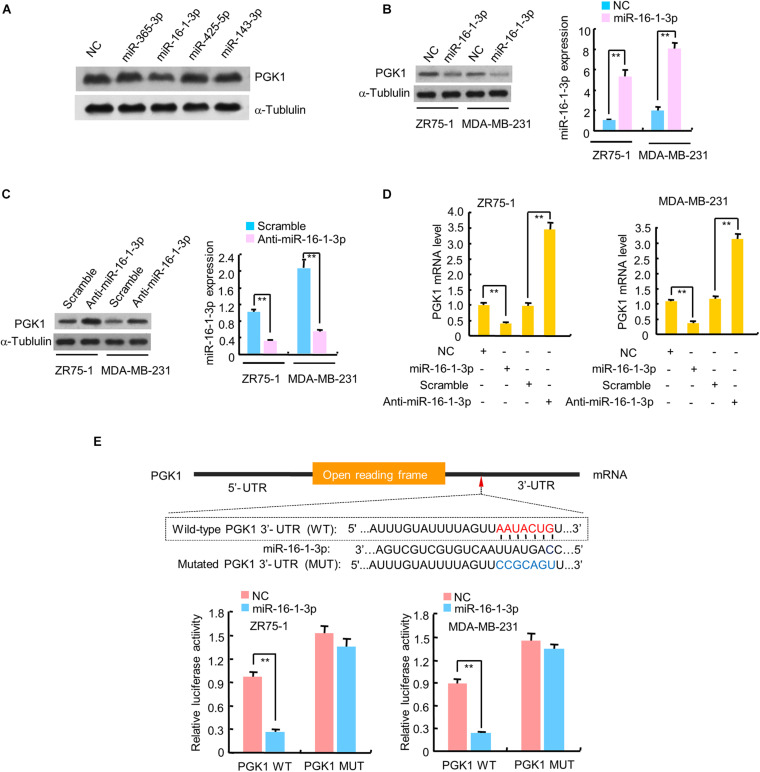
miR-16-1-3p suppresses PGK1 expression by directly targeting its 3′-UTR in breast cancer cells. **(A)** HEK293T cells were transfected with negative control (NC) for miRNAs or mimics of candidate miRNAs as indicated. The representative immunoblot shows PGK1 expression. α-Tubulin was used as a loading control. **(B,C)** Immunoblot analysis of PGK1 expression in ZR75-1 and MDA-MB-231 breast cancer cells transfected with **(B)** NC or miR-16-1-3p mimics, or **(C)** scramble for miRNA inhibitors or miR-16-1-3p inhibitor (anti-miR-16-1-3p). Right histograms indicate relative miR-16-1-3p expression by RT-qPCR. **(D)** RT-qPCR analysis of PGK1 mRNA expression in ZR75-1 and MDA-MB-231 cells transfected as in panels **(B,C)**. **(E)** miRNA luciferase reporter assays of ZR75-1 and MDA-MB-231 cells transfected with wild-type (WT) or mutated (MUT) PGK1 reporter plus miR-16-1-3p mimics. The top panel indicates WT and MUT forms of putative miR-16-1-3p target sequences of PGK1 3′-UTR. Red font indicates the putative miR-16-1-3p binding sites within human PGK1 3′-UTR. Blue font shows the mutations introduced into the PGK1 3′-UTR. Data shown are mean ± SD of triplicate measurements that were repeated three times with similar results. **P < 0.01.

Since PGK1 is the first ATP-generating enzyme in the glycolysis pathway and plays an important role in the development and progression of cancer, we identified potential miRNAs regulating PGK1 by using two target prediction programs, miRanda^1^ and TargetScan.^2^ Our analysis predicted several potential PGK1-targeting miRNAs, among which only miR-16-1-3p inhibited PGK1 protein expression in human embryonic kidney HEK293T cells and two breast cancer cell lines, ZR75-1 and MDA-MB-231 (Figure 1A and Supplementary Figure 1A). We further performed experiments by increasing or reducing mi-16-1-3p expression in ZR75-1 and MDA-MB-231. As expected, miR-16-1-3p mimics reduced PGK1 protein expression in these cells (Figure 1B), whereas miR-16-1-3p inhibitor (anti-miR-16-1-3p) increased PGK1 protein expression (Figure 1C). Moreover, miR-16-1-3p mimics suppressed PGK1 mRNA expression, while miR-16-1-3p inhibitor enhanced PGK1 mRNA expression (Figure 1D).

To investigate whether the inhibitory effect of miR-16-1-3p on PGK1 was mediated through direct binding of PGK1 3′-UTR, ZR75-1 and MDA-MB-231 cells were transfected with wild-type PGK1 3′-UTR or mutated PGK1 3′-UTR luciferase reporter and miR-16-1-3p mimics. Overexpression of miR-16-1-3p decreased the wild-type 3′-UTR luciferase activity, but not the reporter activity of mutated 3′-UTR (Figure 1E). Taken together, these results suggest that miR-16-1-3p inhibits PGK1 expression by targeting PGK1 3′-UTR.

### The MiR-16-1-3p/PGK1 Axis Regulates Glycolysis in Breast Cancer Cells

**FIGURE 2:**
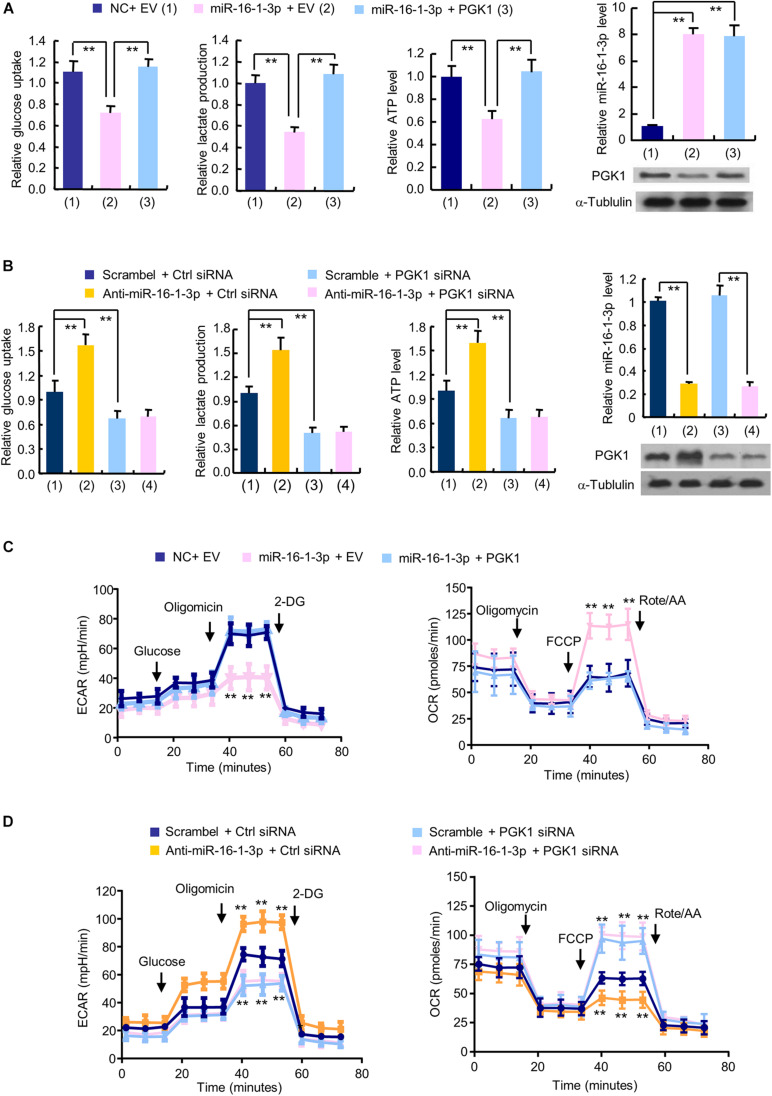
The miR-16-1-3p/PGK1 axis modulates aerobic glycolysis in breast cancer cells. **(A)** Glucose uptake and the production of lactate and ATP were examined in MDA-MB-231 cells transfected with miR-16-1-3p or miR-16-1-3p plus PGK1 expression vector as indicated. EV, empty vector. **(B)** Glucose uptake and the production of lactate and ATP were examined in MDA-MB-231 cells transfected with anti-miR-16-1-3p, PGK1 siRNA or anti-miR-16-1-3p plus PGK1 siRNA. Ctrl siRNA, control siRNA. Representative immunoblot shows PGK1 expression, and RT-qPCR analysis indicates miR-16-1-3p expression **(A,B)**. **(C)** ECAR and OCR assays of MDA-MB-231 cells transfected as in panel **(A)**. **(D)** ECAR and OCR assays of MDA-MB-231 cells transfected as in panel **(B)**. Data shown are mean ± SD of quintuplicate measurements that were repeated three times with similar results [panels **(A,B)** for glucose uptake and the production of lactate and ATP]. Data shown are mean ± SD of triplicate measurements that were repeated three times with similar results (A and B for RT-qPCR analysis). **P < 0.01 **(A,B)**. Data shown are mean ± SD of quintuplicate measurements that were repeated 3 times with similar results **(C,D)**. **P < 0.01 vs NC plus EV or Scramble plus Ctrl shRNA **(C,D)**.

The glycolytic pathway is the main metabolic pathway for tumor cells to perform energy metabolism. In this process, every molecule of glucose taken by cancer cells can quickly generate 2 molecules of ATP to meet their own energy needs. Regardless of whether oxygen is sufficient, the final product of tumor glycolysis is lactate. As PGK1 is a key enzyme in the glycolytic pathway and miR-16-1-3p is an upstream regulator of PGK1, we examined the effect of the miR-16-1-3p/PGK1 axis on aerobic glycolysis. MiR-16-1-3p mimics decreased glucose uptake and production of lactate and ATP in MDA-MB-231 cells (Figure 2A). Reexpression of PGK1 in the miR-16-1-3p-transfected cells reversed these effects. Conversely, anti- miR-16-1-3p increased glucose uptake and production of lactate and ATP (Figure 2B). PGK1 knockdown had opposite effects. Importantly, PGK1 knockdown abolished the ability of anti-miR-16-1-3p to increase glucose uptake and production of lactate and ATP, suggesting that miR-16-1-3p regulates glucose uptake and production of lactate and ATP through PGK1. Moreover, in MDA-MB-231 cells, overexpression of miR-16-1-3p led to a decrease in ECAR, which reflects overall glycolytic flux, and an increase in OCR, an indicator of mitochondrial respiration (Figure 2C). Reexpression of PGK1 in the miR-16-1-3p-transfected cells rescued these effects. In contrast, anti-miR-16-1-3p increased ECAR and decreased OCR in MDA-MB-231 cells (Figure 2D). PGK1 knockdown had opposite effects. Importantly, PGK1 knockdown abrogated the ability of anti-miR-16-1-3p to increase ECAR and decrease OCR, suggesting that miR-16-1-3p regulates ECAR and OCR through PGK1. Similar results were observed in ZR75-1 cells (Supplementary Figures 1B–E). Taken together, these data suggest that miR-16-1-3p regulates glycolysis via PGK1.

### Aerobic Glycolysis Is Responsible for MiR-16-1-3p Modulation of Breast Cancer Cell Proliferation

**FIGURE 3:**
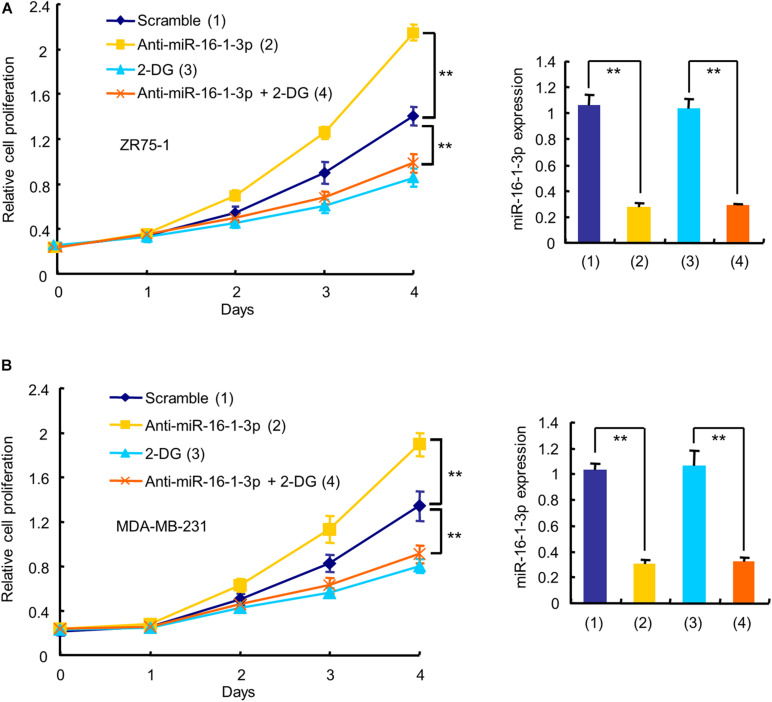
Aerobic glycolysis is responsible for miR-16-1-3p modulation of breast cancer cell proliferation. The proliferation curve shows ZR75-1 **(A)** and MDA-MB-231 **(B)** cells transfected with anti-miR-16-1-3p or scramble and treated with 2.5 mM 2-DG as indicated. RT-qPCR reveals miR-16-1-3p expression. Data shown are mean ± SD of triplicate measurements that were repeated three times with similar results. **P < 0.01.

Since aerobic glycolysis is a hallmark of cancer and the biological function of miR-16-1-3p in breast cancer is unknown, we investigated whether miR-16-1-3p regulates breast cancer cell proliferation and whether aerobic glycolysis plays a role in miR-16-1-3p -mediated regulation of breast cancer cell proliferation. As expected, the glycolytic inhibitor 2-deoxy-D-glucose (2-DG) inhibited proliferation of ZR75-1 and MDA-MB-231 breast cancer cells (Figure 3). Intriguingly, the miR-16-1-3p inhibitor (anti-miR-16-1-3p) promoted breast cancer cell proliferation. 2-DG almost abrogated the ability of anti-miR-16-1-3p to promote breast cancer cell proliferation, suggesting that aerobic glycolysis is responsible for breast cancer cell proliferation regulated by miR-16-1-3p.

### The MiR-16-1-3p/PGK1 Axis Regulates Breast Cancer Cell Proliferation, Migration, and Invasion

**FIGURE 4:**
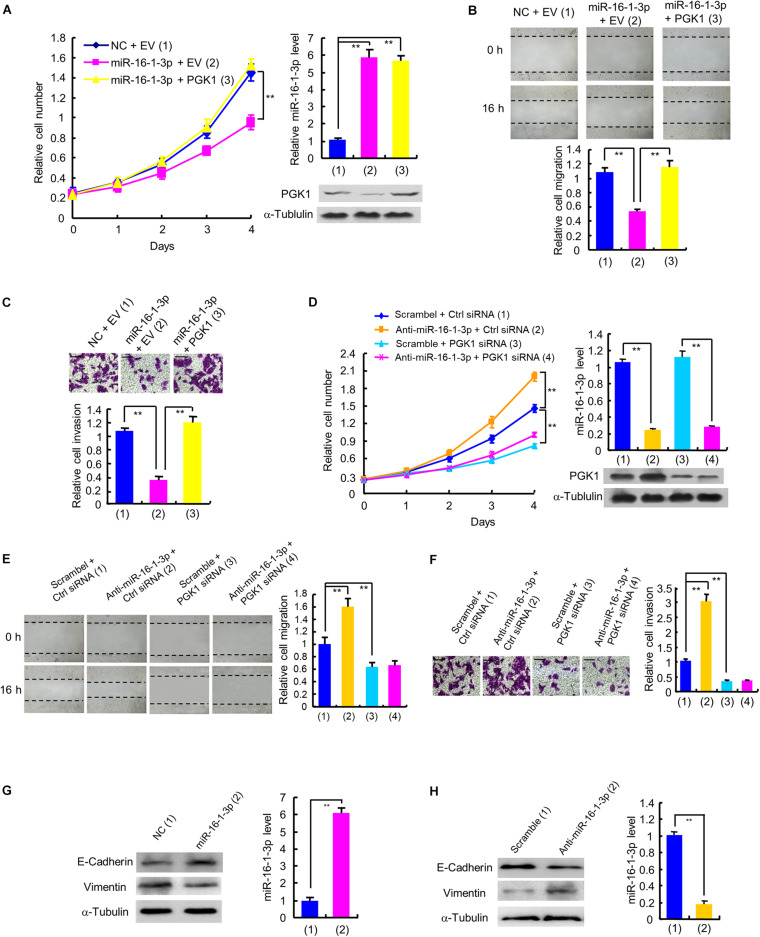
miR-16-1-3p inhibits proliferation, migration and invasion by inhibiting PGK1 expression in breast cancer cells. **(A)** The proliferation curve of MDA-MB-231 cells transfected with miR-16-1-3p mimics or miR-16-1-3p mimics plus PGK1 expression plasmid as indicated. Immunoblot analysis shows PGK1 expression. RT-qPCR indicates miR-16-1-3p expression. **(B,C)** Wound healing **(B)** and invasion **(C)** assays of MDA-MB-231 cells transfected as in panel **(A)**. Histograms denote relative cell migration **(B)** and invasion **(C)**. **(D)** The proliferation curve of MDA-MB-231 cells transfected with anti-miR-16-1-3p, PGK1 siRNA or anti-miR-16-1-3p plus PGK1 siRNA as indicated. Immunoblot analysis shows PGK1 expression. RT-qPCR indicates miR-16-1-3p expression. **(E,F)** Wound healing **(E)** and invasion **(F)** assays of MDA-MB-231 cells transfected as in panel **(D)**. Histograms show relative cell migration **(E)** and invasion **(F)**. All values shown are mean ± SD of triplicate measurements that were repeated three times with similar results. **(G)** Immunoblot analysis of E-Cadherin and Vimentin expression in MDA-MB-231 cells transfected with NC or miR-16-1-3p mimics. **(H)** Immunoblot analysis of E-Cadherin and Vimentin expression in MDA-MB-231 cells transfected with scramble or miR-16-1-3p inhibitor. **P < 0.01.

Next, we examined whether miR-16-1-3p inhibits proliferation, migration and invasion by repressing PGK1 expression in breast cancer cells. Cell proliferation experiments showed that overexpression of miR-16-1-3p suppressed the proliferation of MDA-MB-231 and ZR75-1 cells (Figure 4A and Supplementary Figure 2A). These effects were reversed by PGK1 reexpression in the miR-16-1-3p-transfected cell lines. Overexpression of miR-16-1-3p also revealed reduced migration and invasion ability (Figures 4B,C and Supplementary Figures 2B,C). Again, PGK1 reexpression in the miR-16-1-3p-transfected cells reversed these effects. Conversely, anti-miR-16-1-3p increased breast cancer cell proliferation, migration, and invasion in MDA-MB-231 and ZR75-1 cells (Figures 4D–F and Supplementary Figures 2D–F). PGK1 knockdown had opposite effects. Importantly, PGK1 knockdown abolished the ability of anti-miR-16-1-3p to increase breast cancer cell proliferation, migration, and invasion. Moreover, miR-16-1-3p mimics increased expression of E-cadherin, an epithelial marker, and decreased that of Vimentin, a mesenchymal marker (Figure 4G), while anti-16-1-3p had opposite effects (Figure 4H), suggesting that miR-16-1-3p may regulate epithelial-mesenchymal transition (EMT), a process critical for cancer cell migration and invasion. Taken together, these results suggest that miR-16-1-3p inhibits breast cancer cell proliferation, migration and invasion by repressing PGK1 expression.

### The MiR-16-1-3p/PGK1 Axis Regulates Tumor Glycolysis, Growth and Metastasis in Nude Mice

**FIGURE 5:**
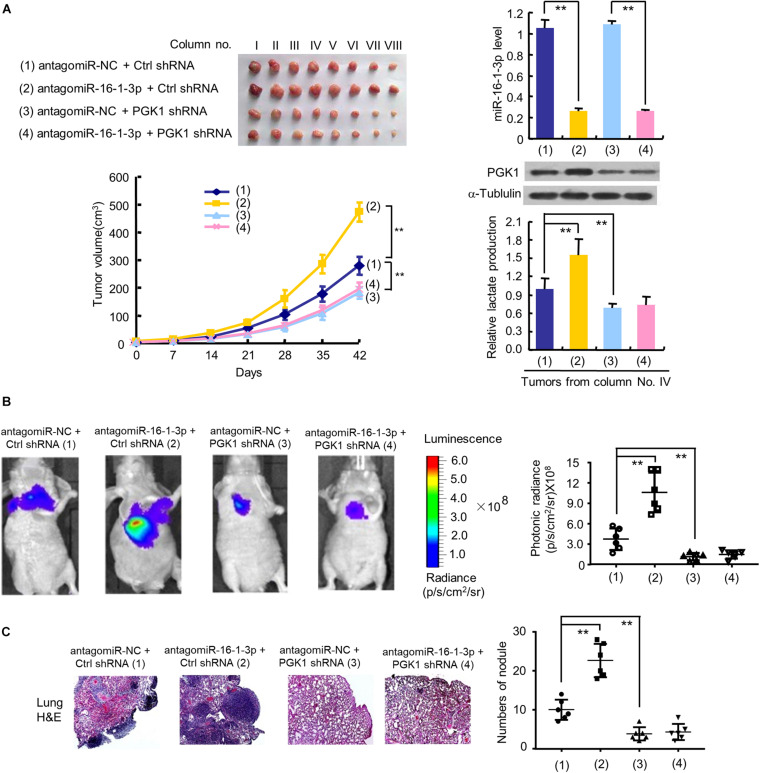
The miR-16-1-3p/PGK1 axis regulates breast tumor growth and metastasis in nude mice. **(A)** MDA-MB-231 cells stably infected with lentivirus harboring PGK1 shRNA or control shRNA (Ctrl shRNA) were treated with antagomiR-16-1-3p or antagomiR-NC and injected into nude mice as indicated. After 42 days, mice were euthanized to harvest tumors. Images of all xenograft tumors excised at day 42 are shown. The tumor growth curves were plotted. Lactate production of representative tumor tissues was measured. miR-16-1-3p and PGK1 expression of representative tumor tissues was determined by RT-qPCR and immunoblot, respectively. Tumor volumes are presented as means ± SD (*n* = 8). **P < 0.01 at day 42. Data shown are mean ± SD of quintuplicate measurements for lactate production that were repeated 3 times with similar results. **P < 0.01. **(B)** Representative bioluminescence images at 30 days of nude mice injected by tail vein with MDA-MB-231 cells expressing firefly luciferase and the indicated constructs (*n* = 6). The luminescence signal is represented by an overlaid false-color image with the signal intensity indicated by the scale **(right panel)**. **(C)** Representative H&E-stained sections of the lung tissues from **(B)**. The number of tumor nodules are shown **(right panel)**. **P < 0.01 **(B,C)**.

To investigate the effect of the miR-16-1-3p/PGK1 axis on breast tumor growth *in vivo*, we used MDA-MB-231 cells harboring anti-miR-16-1-3p or PGK1 shRNA or anti-miR-16-1-3p plus PGK1 shRNA to inject the mammary fat pads of BALB/c nude mice. The results showed that tumor growth was significantly slower in the PGK1 shRNA group and faster in the anti-miR-16-1-3p group than that in the control group (Figure 5A). Moreover, the ability of anti-miR-16-1-3p to promote tumor growth was compromised by PGK1 knockdown. After analyzing lactate production and PGK1 expression of representative tumor tissues, we found that PGK1 expression was stimulated by anti-miR-16-1-3p, and anti-miR-16-1-3p promoted lactate production via PGK1 (Figure 5A). These results suggest that miR-16-1-3p inhibits tumor growth via PGK1-mediated glycolysis.

Next, we explored whether the miR-16-1-3p/PGK1 axis regulates breast cancer metastasis. The results showed that the luminescence signal in the lung region of mice in the anti-miR-16-1-3p group and the PGK1 shRNA group was significantly stronger or weaker, respectively, than that in the control group (Figure 5B). Similar results were observed with the number of nodules in the lung region of mice in the anti-miR-16-1-3p group and the PGK1 shRNA group (Figure 5C). Importantly, the anti-miR-16-1-3p’s stimulatory effect on breast tumor lung metastasis was abolished by PGK1 knockdown (Figures 5B,C). These results suggest that miR-16-1-3p suppresses breast tumor lung metastasis via PGK1.

### Association of MiR-16-1-3p With PGK1 Expression and Metatstasis in Human Breast Cancer Patients

**FIGURE 6:**
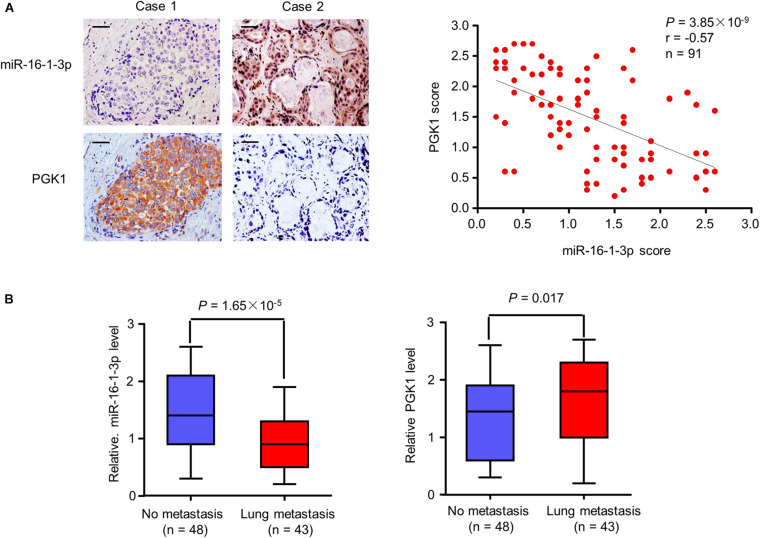
Correlation of miR-16-1-3p expression with PGK1 expression and metastasis in breast cancer patients. **(A)** Correlation between miR-16-1-3p and PGK1 expression in 91 breast cancer patients. miR-16-1-3p and PGK1 expression was assessed by MISH and IHC, respectively. Scale bar: 100 μm. The *P* value was generated using Spearman’s Rank Correlation test. **(B)** Association of miR-16-1-3p expression with metastasis. The *P* value was generated using independent *t* test.

Since miR-16-1-3p inhibits PGK1 expression and suppresses breast cancer lung metastasis, we evaluated the expression of miR-16-1-3p and PGK1 by MISH and IHC, respectively, in 91 human breast cancer samples. Consistent with miR-16-1-3p inhibition of PGK1 *in vitro* and in mice, there was a negative correlation between miR-16-1-3p and PGK1 expression in breast cancer patients (Figure 6A). Moreover, miR-16-1-3p expression negatively correlated with breast cancer lung metatstasis, and PGK1 expression positively associated with breast cancer lung metatstasis (Figure 6B). The specificity of the anti-PGK1 antibody and the miR-16-1-3p probe used was confirmed (Supplementary Figure 3). In addition, miR-16-1-3p negatively correlated with tumor size, nodal status, and grade (Supplementary Table 1), and PGK1 positively correlated with tumor size, nodal status, and grade (Supplementary Table 2). Breast cancer patients with decreased miR-16-1-3p expression had shorter disease-free survival (Supplementary Figure 4A), and breast cancer patients with decreased PGK1 expression had longer disease-free survival (Supplementary Figure 4B). Taken together, these data suggest that the miR-16-1-3p/PGK1 axis plays an important pathological role in human breast cancer.

On the other hand, we compared miR-16-1-3p expression between breast cancer tissues and matched normal tissues from TCGA (The Cancer Genome Atlas) dataset. Surprisingly, miR-16-1-3p expression was upregulated in breast cancer tissues (Supplementary Figure 4C). However, miR-16-1-3p expression was downregulated in plasma of breast cancer patients from GSE58606 dataset (Supplementary Figure 4D). Thus, the clinical significance of miR-16-1-3p in breast cancer needs further validation. Furthermore, there was no correlation between miR-16-1-3p expression and PGK1 mRNA expression in 764 cases of infiltrating ductal carcinoma from TCGA dataset (Supplementary Figure 4E). The discrepancy in the correlation between results of our study and those from the TCGA dataset may be that PGK1 expression was examined in this study at the protein level but not at the mRNA level shown in the TCGA dataset, and only ductal breast carcinoma was detected in the TCGA dataset, while besides ductal breast carcinoma, other breast cancer types such as lobular breast carcinoma were also examined in this study.

## Discussion

In recent years, the relationship between tumor glucose metabolism and anti-tumor therapy has become a research hotspot. One of the salient features of tumor metabolism is reprogramming of energy metabolism, which is manifested by abnormal glucose metabolism. Even when oxygen is sufficient, tumor cells still tend to undergo glycolysis and metabolize glucose to lactic acid. The aerobic glycolytic capacity of tumor cells is 20–30 times that of normal cells, which provides a lot of energy and intermediate products for tumor metabolism. Thus, targeting metabolic enzymes in abnormal metabolic pathways such as glycolytic pathway has become the focus of anti-tumor therapy (Gill et al., 2016; Abdel-Wahab et al., 2019; Kim, 2019; Weiss, 2020). Researchers are exploring the abnormal regulation of glucose metabolism in tumors and the inhibitors targeting related metabolic responses so as to facilitate better treatment and prognosis for tumor patients by inhibiting tumor glycolysis or switching to normal metabolic pathways.

Phosphoglycerate kinase 1 is one of the key enzymes in tumor glucose metabolism. High PGK1 expression was shown in various human cancers and correlated with poor prognosis of cancer patients (Ahmad et al., 2013; Hu et al., 2017; Fu et al., 2018). Phosphoglycerate kinase 1 promotes cancer cell proliferation, invasion and metastasis (Zhang et al., 2019, 2020; Fu and Yu, 2020). Because of this, scientists are working hard to explore PGK1 inhibitors. Very recently, miRNA-450b-3p, miR-548c-5p, miRNA-215-5p, and miR-6869-5p have been reported to directly target PGK1 3′-UTR and repress PGK1 expression (Chen et al., 2019, 2020; Ge et al., 2019; Wang et al., 2020). However, it remains unknown whether these miRNAs regulate the Warburg effect and whether the Warburg effect is responsible for cancer cell growth by these miRNAs. In this study, we discovered for the first time that miR-16-1-3p is another PGK1 inhibitor, and can modulate cancer cell growth and metatstasis by a variety of biological function tests. Importantly, we revealed that in human breast cancer, miR-16-1-3p can inhibit tumor glycolysis by repressing glucose uptake and lactate production, with decreased ECAR and increased OCR. MiR-16-1-3p plays an important role in regulation of PGK1-mediated Warburg effect and breast cancer cell proliferation, migration, invasion, and metastasis. In breast cancer specimens, miR-16-1-3p expression is inversely correlated with PGK1 expression. Therefore, miR-16-1-3p activation may have a positive effect on the treatment of breast cancer patients with PGK1 overexpression.

MiRNAs have been shown to play an important role in cancer development and progression. However, the biological functions of miR-16-1-3p in cancer are largely unknown. MiR-16-1-3p was shown to act as a tumor suppressor (Wang et al., 2017). MiR-16-1-3p negatively regulates transcription factor Twist1 to inhibit NSCLC cell migration and invasion (Feng et al., 2018). MiR-16-1-3p has powerful tumor suppressive and anti-metastatic properties in osteosarcoma (Maximov et al., 2019). Except for gastric cancer, osteosarcoma and lung cancer, the biological function of miR-16-1-3p in other cancers remains unclear. In this study, we showed that miR-16-1-3p represses breast cancer cell proliferation, migration, invasion, and metastasis. MiR-16-1-3p expression was downregulated in breast cancer samples we collected. Cancer cells inhibit miRNA expression via various factors, such as methylation of CpG islands in miRNA promoters, transcription factors, long non-coding RNAs (lncRNAs), etc. For example, CpG islands of miR-137 promoter are hypermethylated in endometrial cancers (Zhang W. et al., 2018), resulting in lower miR-137 expression. The transcription factor c-Myc represses miR-129-5p expression in hepatocellular carcinoma (Han et al., 2016). Overexpression of lncRNA CASC11 promotes bladder cancer cell proliferation via inhibition of miR-150 expression (Luo et al., 2019). The mechanisms by which miR-16-1-3p is repressed in breast cancer require to be investigated. Moreover, we also used *in vitro* and *in vivo* experiments to tightly combine the Warburg effect with cancer cell proliferation, migration and invasion regulated by miR-16-1-3p, enriching the theory of tumor glycolysis at the molecular level.

## Conclusion

Our study revealed for the first time that miR-16-1-3p inhibits tumor glycolysis by targeting PGK1 both *in vitro* and *in vivo*, thereby repressing breast cancer cell proliferation, migration, invasion, and metastasis. In breast cancer patients, the abundance of miR-16-1-3p is inversely related to PGK1 expression. Therefore, up-regulating miR-16-1-3p may provide new treatment ideas for cancer patients, especially breast cancer patients with PGK1 overexpression.

## Data Availability Statement

The raw data supporting the conclusions of this article will be made available by the authors, without undue reservation.

## Ethics Statement

The studies involving human participants were reviewed and approved by the Ethics Committee of the Chinese PLA General Hospital. The patients/participants provided their written informed consent to participate in this study. The animal study was reviewed and approved by the Institutional Animal Care and Use Committee of Beijing Institute of Biotechnology.

## Author Contributions

XZ conceived the project, designed the study, and analyzed the data. TY designed and performed the experiments and analyzed the data, aided by YL and DZ. TY and XZ wrote the manuscript. All authors contributed to the interpretation of the data and approved the content of the manuscript.

## Conflict of Interest

The authors declare that the research was conducted in the absence of any commercial or financial relationships that could be construed as a potential conflict of interest.

## Footnotes

1 http://www.microrna.org/

2 http://www.targetscan.org/
