# Corrigendum: MicroRNA-16-1-3p Represses Breast Tumor Growth and Metastasis by Inhibiting PGK1-Mediated Warburg Effect

**DOI:** 10.3389/fcell.2021.649787

**Published:** 2021-01-21

**Authors:** Tianxing Ye, Yingchun Liang, Deyu Zhang, Xuewu Zhang

**Affiliations:** ^1^College of Medicine, Yanbian University, Yanji, China; ^2^Department of Medical Molecular Biology, Beijing Institute of Biotechnology, Collaborative Innovation Center for Cancer Medicine, Beijing, China

**Keywords:** the Warburg effect, PGK1, miR-16-1-3p, cell proliferation, metastasis

An author name was incorrectly spelled as **Tianxin Ye**. The correct spelling is **Tianxing Ye**.

The authors apologize for this error and state that this does not change the scientific conclusions of the article in any way. The original article has been updated.

