# Supplementary material for: MicroRNA-16-1-3p Represses Breast Tumor Growth and Metastasis by Inhibiting PGK1-Mediated Warburg Effect
Source: Front Cell Dev Biol. 2020 Dec 3;8:615154. doi: 10.3389/fcell.2020.615154 (PMC7744604; doi:10.3389/fcell.2020.615154)
Supplement: Supplementary file 1 [file Data_Sheet_1.PDF]

Figure S1

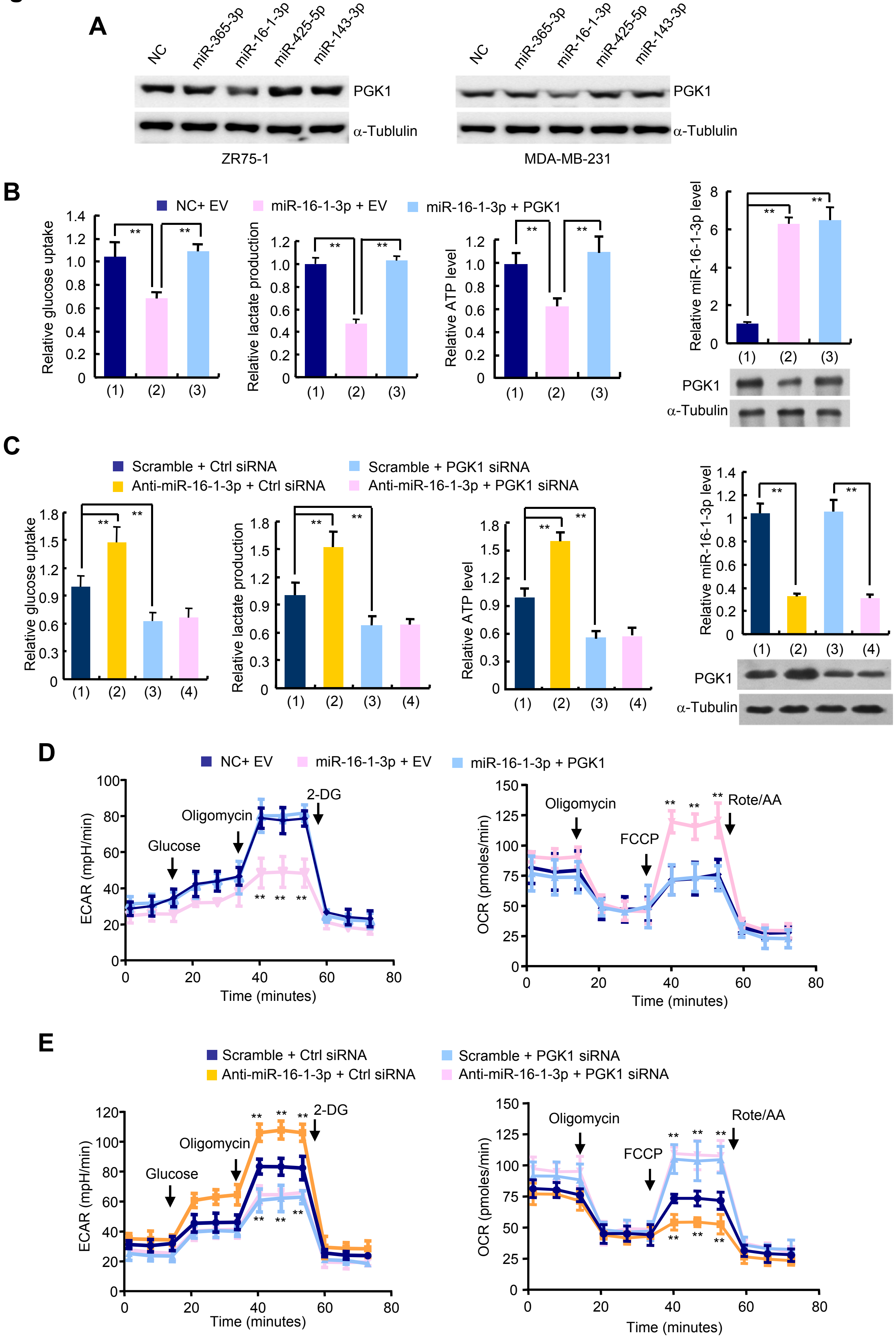

A
NC
miR-365-3p
miR-16-1-3p
miR-425-5p
miR-143-3p
PGK1
α-Tublulin
ZR75-1
MDA-MB-231
B
NC+ EV
miR-16-1-3p + EV
miR-16-1-3p + PGK1
Relative glucose uptake
Relative lactate production
Relative ATP level
Relative miR-16-1-3p level
(1)
(2)
(3)
PGK1
α-Tubulin
C
Scramble + Ctrl siRNA
Anti-miR-16-1-3p + Ctrl siRNA
Scramble + PGK1 siRNA
Anti-miR-16-1-3p + PGK1 siRNA
Relative glucose uptake
Relative lactate production
Relative ATP level
Relative miR-16-1-3p level
(4)
PGK1
α-Tubulin
D
NC+ EV
miR-16-1-3p + EV
miR-16-1-3p + PGK1
ECAR (mpH/min)
Glucose
Oligomycin
2-DG
Time (minutes)
OCR (pmoles/min)
FCCP
Rote/AA
E
Scramble + Ctrl siRNA
Anti-miR-16-1-3p + Ctrl siRNA
Scramble + PGK1 siRNA
Anti-miR-16-1-3p + PGK1 siRNA
ECAR (mpH/min)
OCR (pmoles/min)
Time (minutes)

Supplementary Figure S1 The miR-16-1-3p/PGK1 axis modulates aerobic glycolysis in ZR75-1 cells. **(A)** ZR75-1 and MDA-MB-231 cells were transfected with negative control or mimics of candidate miRNAs as indicated. The representative immunoblot shows PGK1 expression. α-Tubulin was used as a loading control. **(B)** Glucose uptake and the production of lactate and ATP were examined in ZR75-1 cells transfected with miR-16-1-3p or miR-16-1-3p plus PGK1 expression vector as indicated. NC, negative control for miRNA. EV, empty vector. **(C)** Glucose uptake and the production of lactate and ATP were examined in ZR75-1 cells transfected with anti-miR-16-1-3p, PGK1 siRNA or anti-miR-16-1-3p plus PGK1 siRNA. Scramble, negative control for anti-miRNA. Ctrl siRNA, control siRNA. Representative immunoblot shows PGK1 expression, and RT-qPCR analysis indicates miR-16-1-3p expression (B and C). **(D)** ECAR and OCR assays of ZR75-1 cells transfected as in (B). **(E)** ECAR and OCR assays of ZR75-1 cells transfected as in (C). Data shown are mean ± SD of quintuplicate measurements that were repeated 3 times with similar results (B and C for glucose uptake and the production of lactate and ATP). Data shown are mean ± SD of triplicate measurements that were repeated 3 times with similar results (B and C for RT-qPCR analysis). $^{**}P < 0.01$ (B and C). Data shown are mean ± SD of quintuplicate measurements that were repeated 3 times with similar results (D and E). $^{**}P < 0.01$ vs NC plus EV or Scramble plus Ctrl shRNA (D and E).

Figure S2

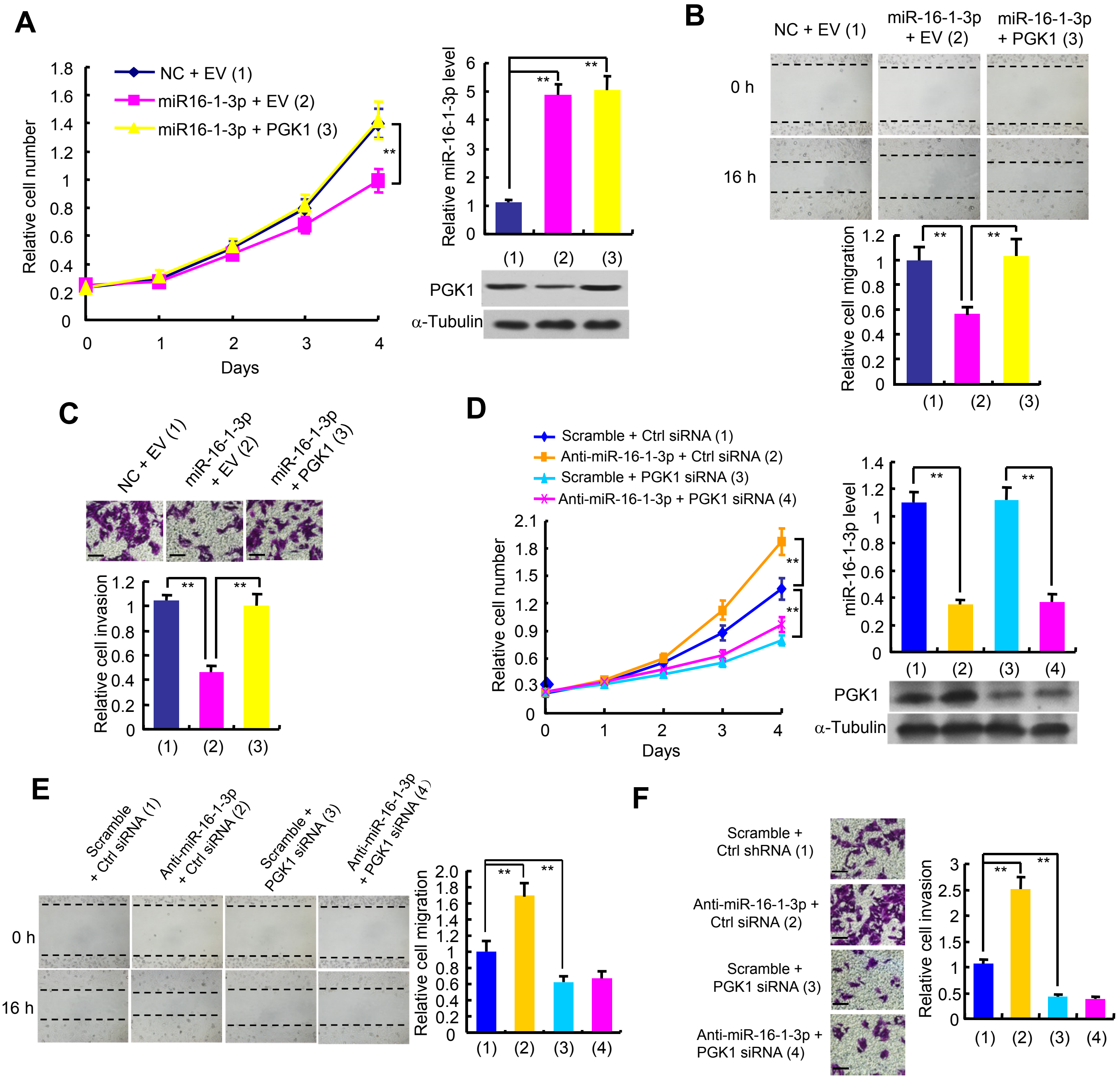

Supplementary Figure S2 miR-16-1-3p inhibits proliferation, migration and invasion by suppressing PGK1 expression in ZR75-1 cells. **(A)** The proliferation curve of ZR75-1 cells transfected with miR-16-1-3p or miR-16-1-3p plus PGK1 expression plasmid as indicated. Immunoblot analysis shows PGK1 expression. RT-qPCR indicates miR-16-1-3p expression. **(B, C)** Wound healing (**B**) and invasion (**C**) assays of ZR75-1 cells transfected as in (**A**). Histograms denote relative cell migration (**B**) and invasion (**C**). **(D)** The proliferation curve of ZR75-1 cells transfected with anti-miR-16-1-3p, PGK1 siRNA or anti-miR-16-1-3p plus PGK1 siRNA as indicated. Immunoblot analysis shows PGK1 expression. RT-qPCR indicates miR-16-1-3p expression. **(E, F)** Wound healing (**E**) and invasion (**F**) assays of ZR75-1 cells transfected as in (D). Histograms show relative cell migration (**E**) and invasion (**F**). All values shown are mean ± SD of triplicate measurements that were repeated 3 times with similar results. $^{**}P < 0.01$.

Figure S3

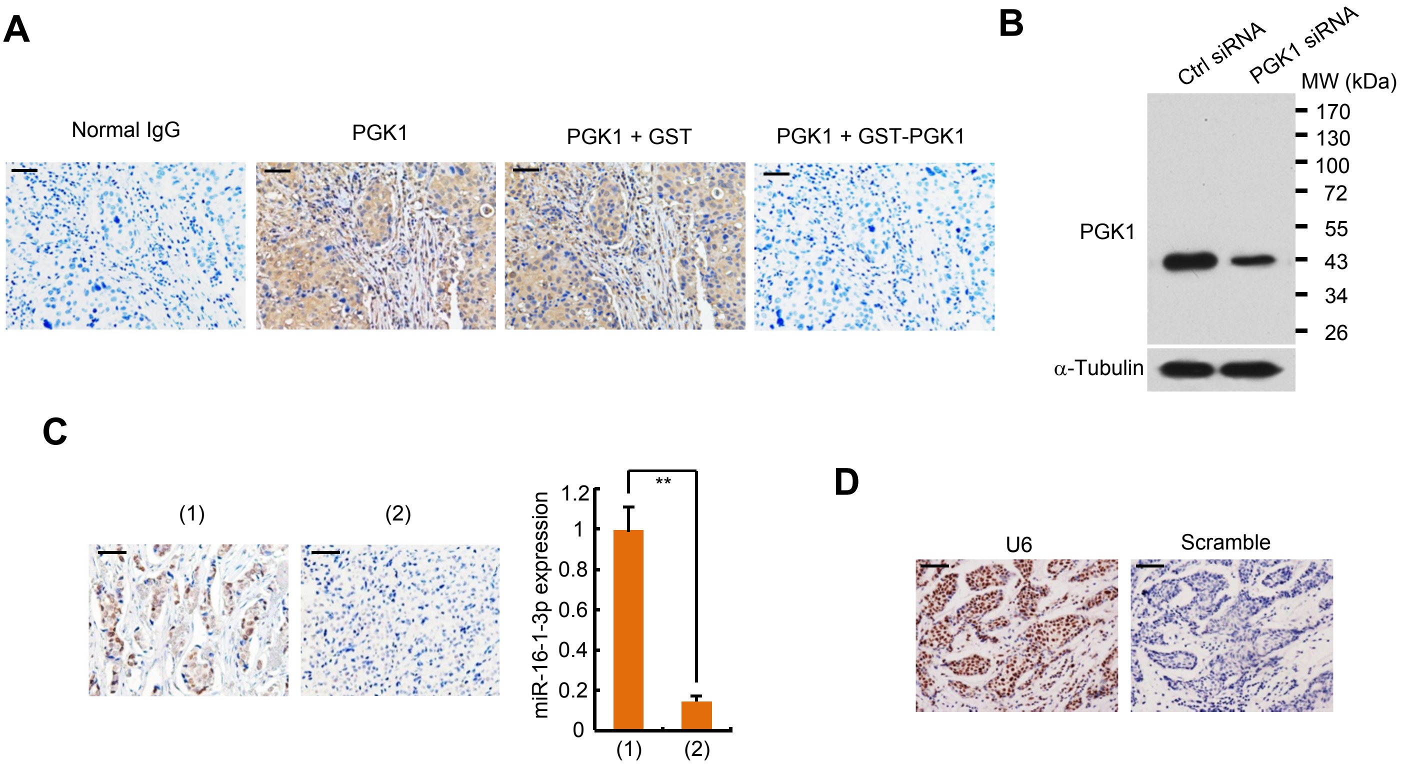

Supplementary Figure S3 Validation of the specificity of an anti-PGK1 antibody and a miR-16-1-3p probe. (**A**) Immunohistochemical staining of representative breast cancer samples incubated with normal IgG or anti-PGK1. To validate antibody specificity, the anti-PGK1 was pre-incubated with recombinant GST-PGK1 protein or GST for 1 h prior to applying to tissue. Scale bar, 50 μm. (**B**) Immunoblot analysis of lysates from ZR75-1 cells transfected with Ctrl siRNA or PGK1 siRNA using antibodies specific for anti-PGK1. MW, molecular weight. (**C**) Different miR-16-1-3p expression levels in 2 different breast cancer tissues examined by MISH (left panel) were confirmed by RT-qPCR (right panel). Scale bar, 50 μm. Data shown are mean ± SD of triplicate measurements that were repeated 3 times with similar results. **$P < 0.01$. (**D**) Positive control (U6) and negative control (Scramble) from the MISH kit were confirmed. Scale bar, 50 μm.

Figure S4

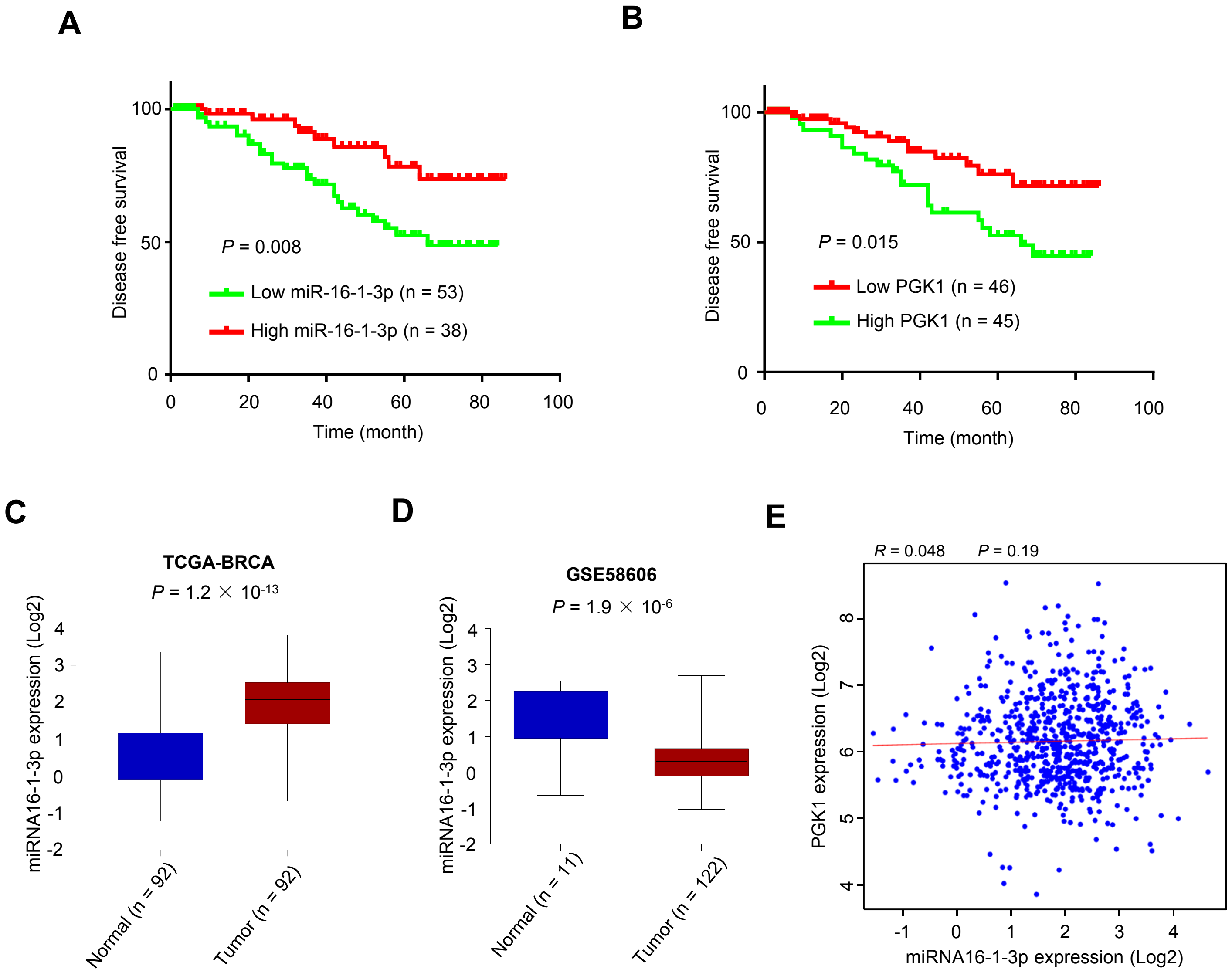

Supplementary Figure S4 Clinical significance of PGK1 and miR-16-1-3p expression in breast cancer. (A, B) The disease-free survival curves related to low and high expression of miR-16-1-3p (A) and PGK1 (B) were analyzed in 91 breast cancer patients using the Kaplan-Meier method. (**C**) Analysis of miR-16-1-3p expression in breast cancer tissues and normal tissues from TCGA-BRCA dataset. (**D**) Analysis of miR-16-1-3p expression in plasma of breast cancer patients from GSE58606 dataset. (**E**) Analysis of correlation of miR-16-1-3p expression with PGK1 mRNA expression of 746 cases of infiltrating ductal carcinoma from TCGA-BRCA dataset.

Supplementary Table 1. Correlations between miR-16-1-3p status and clinicopathologic factors.

Clinical characteristics	Total cases	miR-16-1-3p (Low)	miR-16-1-3p (High)	P value*
Age (years)				
≤ 50	42	27	15	0.279
>50	49	26	23	
Tumor size				
≤ 20 mm	28	11	17	0.019
>20 ≤50 mm	40	24	16	
> 50 mm	23	18	5	
Nodal status				
N0	37	13	24	2.16×10^{-4}
N1+N2+ N3	54	40	14	
Grade				
Grade I	20	7	13	0.041
Grade Ⅱ	32	19	13	
Grade III	39	27	12	
ERα				
Negative	32	22	10	0.134
Positive	59	31	28	
PR				
Negative	41	24	17	0.959
Positive	50	29	21	
HER2				
Negative	61	39	22	0.116
Positive	30	14	16	

**P* value was generated by the chi-quare method.

Supplementary Table 2. Correlations between PGK1 status and clinicopathologic factors.

Clinical characteristics	Total cases	PGK1 (Low)	PGK1 (High)	*P* value*
Age (years)				
≤ 50	42	19	23	0.871
>50	49	23	26	
Tumor size				
≤ 20 mm	28	23	5	1.42×10^{-4}
>20 ≤50 mm	40	17	23	
> 50 mm	23	6	17	
Nodal status				
N0	37	27	10	3.98×10^{-4}
N1+N2+ N3	54	19	35	
Grade				
Grade I	20	16	4	0.003
Grade Ⅱ	32	17	15	
Grade III	39	13	26	
ERα				
Negative	32	14	18	0.339
Positive	59	32	27	
PR				
Negative	41	20	21	0.760
Positive	50	26	24	
HER2				
Negative	61	27	34	0.087
Positive	30	19	11	

**P* value was generated by the chi-quare method.
